# Peer support and reminiscence therapy for people with dementia and their family carers: a factorial pragmatic randomised trial

**DOI:** 10.1136/jnnp-2016-313736

**Published:** 2016-08-12

**Authors:** Georgina Charlesworth, Karen Burnell, Nadia Crellin, Zoe Hoare, Juanita Hoe, Martin Knapp, Ian Russell, Jennifer Wenborn, Bob Woods, Martin Orrell

**Affiliations:** 1 UCL Research Department of Clinical, Educational and Health Psychology, London, UK; 2 School of Health Sciences and Social Work, University of Portsmouth, Portsmouth, UK; 3 North East London NHS Foundation Trust, Ilford, UK; 4 NWORTH Clinical Trials Unit, Bangor University, Bangor, UK; 5 Division of Psychiatry, University College London, London, UK; 6 London School of Economics, London, UK; 7 Swansea University Medical School, Swansea, UK; 8 Division of Psychiatry, University College London, London, UK; 9 DSDC Wales, Bangor University, Bangor, UK; 10 Institute of Mental Health, University of Nottingham, Nottingham, UK

## Abstract

**Objective:**

The objective of this study was to evaluate peer support and reminiscence therapy, separately and together, in comparison with usual care for people with dementia and their family carers.

**Design:**

Factorial pragmatic randomised trial, analysed by treatment allocated, was used for this study.

**Setting:**

The trial ran in Community settings in England.

**Participants:**

People with dementia and their family carers were the participants.

**Interventions:**

Treatment as usual (TAU) plus one of the following: one-to-one peer support to family carers from experienced carers (Carer Supporter Programme; CSP), group reminiscence therapy (Remembering Yesterday, Caring Today; RYCT) for people with dementia and carers, both or neither.

**Main outcome measures:**

Primary outcomes included health-related quality of life (SF-12) for carers and quality of life (QoL-AD) for people with dementia; secondary outcomes included quality of relationship for carers and people with dementia; both were collected by blinded assessors at baseline, 5 and 12 months (primary end point).

**Results:**

Of 291 pairs recruited, we randomised 145 (50%) to CSP (71% uptake) and 194 (67%) to RYCT (61% uptake). CSP and RYCT, separately or together, were not effective in improving primary outcomes or most secondary outcomes. For CSP versus ‘no CSP’, adjusted difference in means was 0.52 points on the SF-12 (95% CI −1.28 to 2.32) and −0.08 points on the QoL-AD (95% CI −1.70 to 1.56). For RYCT versus ‘no RYCT’, the difference was 0.10 points on the SF-12 (95% CI −1.72 to 1.93) and 0.51 points on the QoL-AD (95% CI −1.17 to 2.08). However, carers reported better relationships with the people with dementia (difference 1.11, 95% CI 0.00 to 2.21, p=0.05). Comparison of combined intervention with TAU, and of intervention received, suggested differential impacts for carers and persons with dementia.

**Conclusions:**

There is no evidence from the trial that either peer support or reminiscence is effective in improving the quality of life.

**Trial registration number:**

ISRCTN37956201; Results.

## Introduction

The lack of progress in new drug treatments for people with dementia has contrasted with recent advances in psychological interventions. In the UK, there have been large randomised controlled trials of cognitive–behavioural therapy (CBT)^1^ and befriending^2^ as interventions for carers, cognitive stimulation therapy (CST)^3^ for people with dementia and reminiscence therapy^4^ for carers and people with dementia. The evaluations of CBT for carers and CST for people with dementia have demonstrated the effectiveness of these interventions in reducing distress and maintaining cognition, respectively. In contrast, there is no evidence that befriending^2^ or joint reminiscence^4^ reduces distress, even though they are recommended in clinical guidelines^5^ and remain popular in practice. Peer support may be more effective than befriending in addressing carers' needs and may also enhance reminiscence therapy. Hence, this trial aimed to evaluate two psychological interventions, namely one-to-one peer support for carers (Carer Supporter Programme; CSP) and group reminiscence therapy for people with dementia and their carers (Remembering Yesterday Caring Today; RYCT), together and separately.

## Methods

The trial protocol^6^ describes the research question, sample size calculation, recruitment, consent, randomisation, interventions, outcome measures, ethical considerations and research governance.

### Design

We used a multisite, 2×2 factorial, pragmatic randomised trial. The factorial design in effect is ‘two trials in one’, enabling us to evaluate both treatments. We consented participating carers, collected baseline data and randomised them individually between CSP and treatment as usual (TAU), and then randomised them between RYCT groups and TAU. This created four arms—CSP, RYCT, CSP and RYCT, and TAU alone. To yield enough participants to run viable RYCT groups, randomisations between TAU, CSP, RYCT and CSP–RYCT were in the proportions of 1:2:1:2. At the first randomisation, we stratified by kinship (whether carers were spouses or offspring) and centre. At the second randomisation, we also stratified by the first allocation to keep the four arms in balance. We collected follow-up data 5 and 12 months (main end point) after the first randomisation.

In accordance with MRC guidance on developing and evaluating complex interventions,^7^ we piloted in two London boroughs before the full trial: the first borough reviewed the appropriateness and acceptability of procedures, and the second borough checked logistics and timing of the interventions. We described the resulting decisions on pooling pilot and main trial data elsewhere.^8^ As changes between pilot and full trial were minimal, we carried pilot data forward to the full trial.

### Ethics approval

The Outer North East London Research Ethics Committee gave written ethics approval (09/H0701/54). We obtained Research and Development approval from local trusts. All participants gave written informed consent.

### Setting

The trial ran in community settings in London, Berkshire, Norfolk and Northamptonshire.

### Eligibility criteria

Participants were adult (18 years and over) family carers and their relative with dementia (as defined by DSM-IV criteria) living at home in the community. We excluded carers if they or their protégés had learning disability, non-progressive brain injury or diagnosed terminal illness, or they were already taking part in another psychosocial intervention study.

### Sample size

We based sample size on the BECCA^2^ and REMCARE^4^ trials. These predicted effect sizes, namely average effect per participant divided by population SD, of 0.42 for CSP and 0.35 for RYCT. Hence, a completed sample of 240 pairs would yield power of more than 90% to detect these effects using a significance level of 5%. This would also yield power of more than 80% to detect interaction between CSP and RYCT equivalent to an effect size of 0.4. Assuming 80% retention, we aimed to recruit 300 pairs of carers and people with dementia.

### Interventions

For CSP, volunteer carer supporters (CSs) were recruited locally by CS Coordinators and attended a mandatory ‘Being a Carer Supporter’ orientation and awareness course before being matched with a family carer participant. The target ‘dose’ was 12 weekly meetings of 1 hour, followed by fortnightly meetings for the next 5 months, 22 hours in total. Meetings took place in the carer's own home, or a public venue like a café, or over the telephone, and could include or exclude the person with dementia according to the preference of the family carer. We asked CSs to listen, encourage and give moral support. Though they could also signpost to resources and services, we instructed them not to offer tangible support, respite or direct advice.

The RYCT intervention followed Schweitzer and Bruce's^9^ programme. Twelve weekly sessions took place in community venues, each lasting 2 hours, covered themes across the lifespan, using multisensory triggers and activities, such as group discussions, small group activity, handling objects, acting or improvisation, and singing. During four sessions, the family carers met separately from the main group for ∼45 min with the aim of developing listening and communication skills, and considering how the activities and strategies in the sessions could continue in the home. After the weekly sessions, monthly sessions continued for 7 months, giving a possible 19 sessions over 10 months. To address the potential time burden of the combined intervention, we invited CSs to meet their matched carers at the RYCT sessions.

We had a planned protocol for assessing intervention delivery and receipt.^10^ Delivery of CSP was monitored by local CS Coordinators through monthly completion of checklists, and RYCT adherence was recorded for each session by a participating research assistant. We ensured that all participants continued to receive the TAU available in their area and gave them lists of useful local resources.

### Measures

We collected demographic information for carer and person with dementia, including age, gender, education, kinship and living circumstances. We characterised carers' social networks according to the Practitioner Assessment of Network Typology (PANT).^11^ We measured the cognitive status of the person with dementia by the mini-mental state examination (MMSE)^12^ and the interviewer rated the clinical dementia rating (CDR).^13^

#### Primary outcomes

The primary outcome was family carers' mental health-related quality of life, measured by the mental component score of the UK Short Form-12 Health Survey (UK SF-12).^14^ ^15^ The SF-12 covers physical functioning, social functioning, role functioning (physical and mental), vitality, bodily pain, mental health and general health, and it generates mental and physical component scores (MCS-12 and PCS-12, respectively).

The primary outcome for the person with dementia was quality of life measured by the 13-item Quality of Life—Alzheimer's Disease Scale (QoL-AD).^16^ Responses for both versions—completed by self or proxy—range from poor (1), through fair and good to excellent (4), yielding totals in the range between 13 and 52.

#### Secondary outcomes for family carers

Health-related quality of life using the EQ-5D^17^ comprising five items generating a single utility score and a visual analogue scale (VAS) to rate one's general health, Hospital Anxiety and Depression Scale (HADS),^18^ Emotional Loneliness Scale,^19^ Caregiver Distress Scale of the Neuropsychiatric Inventory (NPI-D),^20^ positive scale from the Positive and Negative Affect Schedule (PANAS),^21^ Positive Aspects of Caring (PAC) using the four-item positive aspects subscale from the Carers of Older People in Europe Index (COPE index),^22^ three-item Personal Growth Index (PGI)^23^ and Quality of Caregiver–Patient Relationship (QCPR).^24^

### Secondary outcomes for the person with dementia

These included the EQ-5D, HADS and QCPR, as for family carers. Family carers rated the functional capacity of the person with dementia in activities of daily living using the Alzheimer's Disease Cooperative Study—Activities of Daily Living Inventory (ADCS-ADL).^25^ We also assessed their quality of life by the DEMQOL,^26^ completed by self and carer.

### Blinding

The nature of the interventions prevented us from blinding participants and providers to their allocated group. However, we blinded research interviewers by provided interventions independently of their assessments. After interview, researchers recorded their perceptions of participants' allocation. This showed no evidence of bias due to non-blinded researchers.

### Data management and statistical analysis

We entered data into Infermed's MACRO Electronic Data Capture system for clinical trials. We audited a randomly selected 10% sample of data for each site at each time point, to ensure that the MACRO database was consistent with paper questionnaires. We corrected all errors and inconsistencies, transferred the resulting data to the Statistical Package for the Social Sciences (SPSS V.20) and undertook further checking, notably for out-of-range values.

Where individual items were missing within scales or subscales, we imputed them before calculating scale or subscale scores. We prorated measures with at most 20% of items missing; for example, if one of five items was missing, we imputed this by the mean of the other four items. We also made multiple imputations within time points, unless all measures were missing at that point. Multiple imputations were calculated using a linear regression model taking into account demographic variables (carer gender, centre, carer age, living status for carer outcomes; carer gender, person with dementia gender, centre, carer age, person with dementia age, living status, baseline MMSE and CDR for person with dementia outcomes), treatment group and other scores provided at a given time point. Using a multiple imputation method allowed an assessment of the sensitivity of the data to the imputations used.

The trial statistician (ZH) carried out all statistical analyses, mostly following a predefined analysis plan by ‘treatment allocated’. We estimated the two main effects by comparing CSP (alone or in combination with RYCT) with no CSP (TAU or RYCT alone); and RYCT (alone or in combination with CSP) with no RYCT (TAU or CSP alone), including interaction between CSP and RYCT in each analysis. We used multilevel analysis of covariance with follow-up data as dependent variable and baseline score as covariate. We treated group allocation, gender and kinship as fixed effects, and centre and time as random effects. When a main effect or interaction term was significant, we repeated the analysis by comparing the combined intervention with TAU. Finally, to explore whether ‘treatment received’ could explain these pragmatic analyses by ‘treatment allocated’, we undertook a form of dose–response analysis by adding the numbers of CSP contacts, RYCT sessions attended and RYCT sessions attended by a CS to the model.

### Patient involvement

Psychosocial interventions are a high priority for people with dementia and family carers. Former family carers were involved in the development and delivery of the CS intervention. Results have been shared with study participants through a lay final report and a stakeholder dissemination event.

## Results

### Participant flow

**Figure 1:**
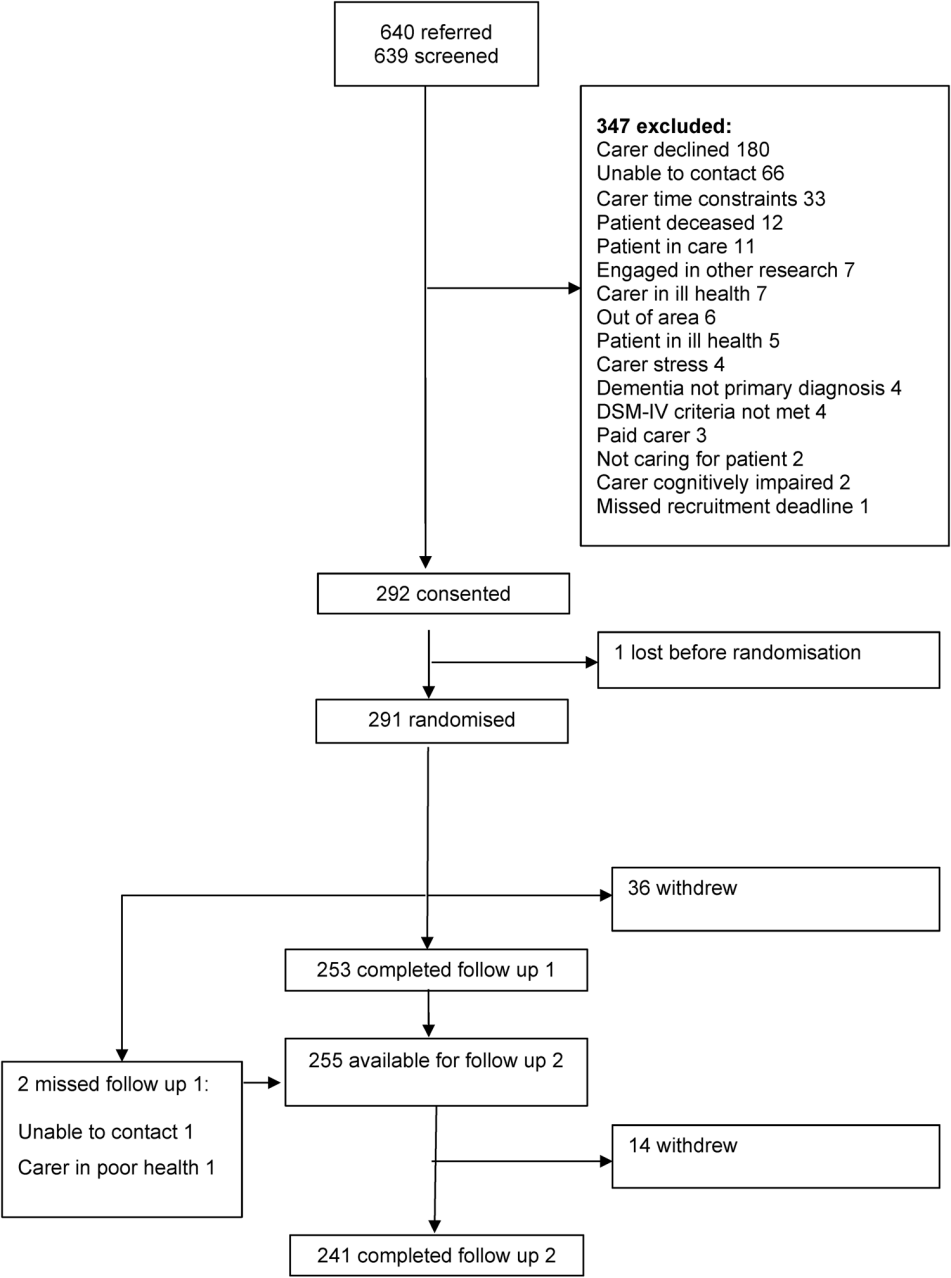
Flow of participants through study.

Figure 1 shows the flow of participants through the trial. The research team received 640 expressions of interest from carers and screened all but one for eligibility. Of these, they excluded 347, notably 180 because carers declined, 66 because researchers could not contact carers and 33 because carer had too little time. Of the 292 family carers who consented to the research, 1 withdrew before randomisation, but the rest completed the baseline assessment and were randomised between January 2010 and March 2012. However, two provided no data at any time point. As we collected no information about potential participants before they consented, we cannot assess whether those who participated differed from those who declined or those whom we excluded from the trial.

Stress, time constraints or the poor health of carer or relative with dementia caused 36 carers to withdraw before the first follow-up after 5 months; 2 of these reappeared for the final follow-up after 12 months. Thus, 253 carers completed the first follow-up. As 241 completed the final follow-up, we retained 83% of the participants through 12 months. Loss to follow-up was greater, but not significantly greater, in the TAU group (23%) than in those allocated to 1 of the 3 interventions (15%).

### Baseline characteristics

Tables 1 and 2 show baseline demographic and clinical characteristics of family carers and people with dementia, respectively. Most family carers were white British (89%), women (67%), spouses (64%) and had had only school education (62%). Their mean age was 67 years, of which they had spent an average of more than 4 years in caring, more than 2 of these since diagnosis. Most people with dementia were white British (88%), women (53%), residing with their carer (68%), and had had only school education (75%). Their mean age was 80 years. Alzheimer's disease was the most common diagnosis, followed by vascular dementia, although a significant proportion had no specific diagnosis recorded.

**Table 1:** Baseline characteristics of family carers by allocated group

		CSP	CSP–RYCT	TAU	RYCT
		(N=48)	(N=97)	(N=47)	(N=97)
Characteristics/measure	Level	n (%)	n (%)	n (%)	n (%)
Gender	Female	29 (64)	66 (68)	30 (64)	72 (74)
Ethnicity	White British	45 (94)	84 (87)	39 (83)	90 (93)
Marital status	Married/cohabiting/civil	44 (92)	85 (88)	37 (79)	82 (85)
Relationship to relative with dementia	Spouse/partner	32 (67)	60 (62)	29 (62)	62 (64)
Living with relative with dementia		39 (81)	78 (80)	40 (85)	73 (75)
Highest level of education	School leaver (aged 14–16)	37 (77)	60 (61)	18 (38)	64 (66)
	Further/higher education	11 (23)	37 (38)	29 (62)	33 (34)
Social network typology	Family dependent	10 (21)	34 (35)	9 (19)	30 (32)
	Locally integrated	13 (27)	32 (33)	16 (34)	29 (31)
	Local self-contained	11 (23)	19 (20)	12 (26)	21 (22)
	Wider community focused	4 (8.3)	4 (4.2)	8 (17)	8 (8.4)
	Private	10 (21)	7 (7.3)	2 (4.3)	7 (7.4)
		**M (SD)**	**M (SD)**	**M (SD)**	**M (SD)**
Age (years)		69.0 (10.5)	65.8 (12.4)	66.8 (14.7)	66.3 (11.8)
Months of caring		58.8 (38.1)	51.2 (42.3)	52.0 (36.2)	50.94 (34.4)
SF12 V.1 (UK)	Mental component*	39.4 (6.04)	39.7 (6.84)	42.5 (6.85)	38.3 (7.50)
	Physical component*	36.9 (7.09)	37.8 (7.687)	39.2 (7.34)	35.8 (8.34)
EQ-5D	Utility*	0.763 (0.191)	0.837 (0.179)	0.726 (0.261)	0.754 (0.255)
	Self-rated global health*	74.4 (21.0)	74.3 (19.8)	63.6 (19.9)	68.1 (20.9)
HADS	Total	12.0 (7.06)	11.7 (7.67)	11.7 (8.81)	13.2 (8.28)
PANAS	Positive affectivity*	31.8 (7.69)	30.5 (6.81)	34.1 (7.96)	29.4 (7.36)
COPE index	PAC*	12.7 (2.16)	12.6 (2.42)	12.7 (2.30)	12.6 (2.20)
Neuropsychiatric inventory	Carer distress	13.5 (8.91)	11.0 (8.63)	11.5 (8.64)	13.4 (13.46)
PGI	Personal growth*	14.5 (3.14)	13.5 (3.36)	15.0 (3.10)	14.0 (2.91)
QCPR	QCPR total*	53.6 (9.22)	52.0 (9.56)	54.9 (8.43)	52.9 (9.28)
Loneliness		2.21 (2.04)	2.41 (2.49)	1.89 (2.36)	2.41 (2.40)

*Higher scores are better.

COPE, Carers of Older People in Europe; EQ-5D, EuroQol 5 dimensions; HADS, Hospital Anxiety and Depression Scale; PAC, Positive Aspects of Caring; PANAS, Positive and Negative Affect Schedule; PGI, Personal Growth Index; QCPR, Quality of Caregiver–Patient Relationship; SF, short form.

**Table 2:** Baseline characteristics of people with dementia by allocated group

		CSP	CSP–RYCT	TAU	RYCT
		(N=48)	(N=97)	(N=47)	(N=97)
Characteristic/measure	Level	n (%)	n (%)	n (%)	n (%)
Gender	Female	27 (56)	49 (51)	29 (62)	48 (49)
Ethnicity	White British	46 (96)	81 (84)	37 (79)	89 (92)
Marital status	Married/cohabiting/civil	34 (71)	68 (70)	29 (62)	65 (67)
Living situation	Living alone	6 (13)	11 (11)	8 (17)	20 (21)
	Cohabiting with partner	35 (73)	65 (67)	29 (62)	65 (67)
	Living with others	7 (15)	21 (22)	7 (15)	10 (10)
Highest level of education	School leaver (aged 14–16)	36 (75)	72 (74)	33 (70)	75 (77)
	Further/higher education	10 (21)	22 (23)	11 (23)	18 (19)
	Missing	2 (4.2)	3 (3.1)	3 (6.4)	4 (4.1)
CDR	0.5–1	26 (57)	57 (60)	32 (73)	61 (73)
	2–3	20 (43)	38 (40)	12 (27)	23 (27)
	Missing	2	2	3	13
		**Mean (SD)**	**Mean (SD)**	**Mean (SD)**	**Mean (SD)**
Age (years)		79.8 (8.18)	79.3 (7.54)	79.5 (7.32)	79.8 (8.36)
Months since diagnosis		29.6 (20.6)	30.9 (25.5)	29.8 (26.1)	33.2 (30.0)
MMSE	Total*	16.3 (6.37)	17.5 (6.35)	19.7 (5.37)	16.3 (7.03)
QoL-AD	Self-report*	37.1 (4.75)	35.6 (5.82)	37.6 (5.94)	36.7 (5.50)
	Proxy*	30.9 (6.01)	30.0 (5.89)	32.2 (6.81)	30.7 (5.35)
DEMQOL	Self-report*	93.4 (12.4)	90.6 (13.4)	92.4 (11.2)	92.1 (12.3)
	Proxy*	89.3 (15.6)	94.4 (14.1)	93.5 (15.8)	93.8 (13.3)
EQ-5D	Utility value*	0.693 (0.312)	0.677 (0.301)	0.763 (0.312)	0.666 (0.316)
	Self-rated general health*	76.2 (18.1)	70.4 (19.4)	76.42 (16.0)	69.60 (21.2)
HADS	Total	8.49 (6.27)	10.6 (6.32)	9.00 (6.28)	9.89 (6.27)
ADCS-ADL	Total*	42.0 (16.4)	41.2 (18.1)	44.3 (19.4)	42.2 (17.6)
NPI	Total	23.4 (17.7)	21.4 (16.9)	22.5 (17.2)	28.1 (22.3)
Quality of relationship	QCPR total*	58.5 (6.11)	57.4 (6.56)	58.6 (5.96)	58.4 (6.41)

*Higher scores are better.

ADCS-ADL, Alzheimer's Disease Cooperative Study—Activities of Daily Living; CDR, clinical dementia rating; DEMQOL, quality of life for people with dementia; EQ-5D, EuroQol 5 dimensions; HADS, Hospital Anxiety and Depression Scale; MMSE, mini-mental state examination; NPI, neuropsychiatric inventory; QCPR, Quality of Caregiver–Patient Relationship; QoL-AD, Quality of Life in Alzheimer's Disease Scale.

Though allocated groups were broadly similar, there were non-significant differences that could have distorted findings if we had not used analysis of covariance, notably people with dementia in the TAU group were less impaired on the MMSE than other groups. To strengthen our analysis, we added MMSE as a covariate for people with dementia. Though ethnicity varied between groups, there was a greater variation between centres, with the proportion of white British carers ranging from 72% to 100%, reflecting the local populations from which we recruited participants.

### Effectiveness

Tables 3 and 4 show the results of analyses after 12 months by treatment allocated for family carers and people with dementia, respectively.

**Table 3:** Outcomes for family carers after 12 months: means and differences adjusted for covariates and the other intervention

Measure	Data set	Missing (N=241)	CSP	No CSP	MD	95% CI of MD	Significance level	RYCT	No RYCT	MD	95% CI of MD	Significance level	Significance level of interaction
SF12 MCS*	Original	0	41.56	41.05	0.517	(−1.28 to 2.32)	0.57	41.36	41.25	0.105	(−1.72 to 1.93)	0.91	0.74
	Imputed	0	41.56	41.05	0.517	(−1.28 to 2.32)	0.57	41.36	41.25	0.105	(−1.72 to 1.93)	0.91	0.74
SF12 PCS*	Original	0	43.86	43.25	0.613	(−1.23 to 2.46)	0.52	43.34	43.77	−0.428	(−2.29 to 1.43)	0.65	0.98
	Imputed	0	43.86	43.25	0.613	(−1.23 to 2.46)	0.52	43.34	43.77	−0.428	(−2.29 to 1.43)	0.65	0.98
EQ-5D utility*	Original	6	0.77	0.72	0.0513	(−0.02 to 0.13)	0.13	0.73	0.76	−0.0250	(−0.10 to 0.05)	0.52	0.79
	Imputed	0	0.77	0.71	0.0574	(−0.02 to 0.13)	0.14	0.72	0.75	−0.0310	(−0.11 to 0.05)	0.43	0.70
EQ-5D self-rated global health*	Original	6	70.72	69.34	1.38	(−3.04 to 5.80)	0.32	70.17	69.90	0.274	(−4.16 to 4.70)	0.87	0.34
	Imputed	0	69.73	69.35	0.384	(−4.20 to 4.96)	0.87	70.00	69.08	0.915	(−3.65 to 5.48)	0.69	0.27
HADS anxiety	Original	3	6.83	7.22	−0.389	(−1.33 to 0.56)	0.22	7.19	6.85	0.336	(−0.61 to 1.28)	0.51	0.38
	Imputed	0	6.91	7.26	−0.33	(−1.31 to 0.60)	0.47	7.23	6.93	0.298	(−0.65 to 1.25)	0.54	0.29
HADS depression	Original	3	5.93	5.96	−0.0254	(−0.90 to 0.85)	0.84	5.93	5.96	−0.0250	(−0.91 to 0.86)	0.94	0.71
	Imputed	0	5.99	5.97	0.0144	(−0.85 to 0.88)	0.97	5.95	6.02	−0.0700	(−0.95 to 0.81)	0.88	0.65
PANAS positive affectivity*	Original	4	30.59	30.37	0.226	(−1.23 to 1.68)	0.77	31.02	29.94	1.07	(−0.40 to 2.56)	0.15	0.93
	Imputed	0	30.47	30.30	0.163	(−1.29 to 1.62)	0.83	30.85	29.92	0.936	(−0.55 to 2.42)	0.22	0.93
COPE PAC*	Original	24	12.14	12.26	−0.116	(−0.64 to 0.41)	0.70	12.26	12.14	0.114	(−0.41 to 0.64)	0.65	0.79
	Imputed	0	12.13	12.11	0.0125	(−0.54 to 0.56)	0.96	12.16	12.08	0.0784	(−0.45 to 0.61)	0.77	0.93
Carer distress NPI-D	Original	37	11.94	9.51	2.435	(0.14 to 4.73)	0.04	10.58	10.87	−0.294	(−2.58 to 1.99)	0.73	0.10
	Imputed	0	12.63	10.82	1.819	(−0.46 to 4.10)	0.12	11.68	11.77	−0.0851	(−2.28 to 2.10)	0.94	0.40
Personal growth*	Original	5	12.03	12.43	−0.396	(−0.98 to 0.19)	0.18	12.41	12.05	0.360	(−0.23 to 0.95)	0.26	0.23
	Imputed	0	12.03	12.44	−0.412	(−1.00 to 0.18)	0.17	12.42	12.05	0.369	(−0.22 to 0.96)	0.22	0.23
QCPR warmth*	Original	24	31.36	31.27	0.0936	(−1.11 to 1.30)	0.82	31.72	30.91	0.819	(−0.40 to 2.04)	0.17	0.69
	Imputed	0	31.32	31.06	0.260	(−0.99 to 1.50)	0.68	31.63	30.74	0.892	(−0.33 to 2.12)	0.15	0.76
QCPR absence of criticism and conflict*	Original	24	22.23	21.45	0.778	(−0.28 to 1.84)	0.15	22.19	21.49	0.704	(−0.35 to 1.76)	0.19	0.97
	Imputed	0	22.08	20.97	1.107	(0.00 to 2.21)	0.05	22.02	21.04	0.981	(−0.15 to 2.11)	0.09	0.76
QCPR total*	Original	24	53.60	52.70	0.903	(−1.03 to 2.83)	0.33	53.89	52.40	1.493	(−0.44 to 3.42)	0.12	0.81
	Imputed	0	53.41	52.00	1.416	(−0.47 to 3.30)	0.14	53.64	51.77	1.869	(−0.02 to 3.75)	0.05	0.84
Loneliness	Original	4	2.68	2.51	0.168	(−0.32 to 0.66)	0.61	2.47	2.71	−0.244	(−0.73 to 0.24)	0.31	0.52
	Imputed	0	2.72	2.53	0.187	(−0.31 to 0.68)	0.46	2.49	2.76	−0.267	(−0.76 to 0.22)	0.29	0.40

Covariates: baseline score, carer gender, kinship and centre (random effect).

*Higher scores are better.

COPE, Carers of Older People in Europe; EQ-5D, EuroQol 5 dimensions; HADS, Hospital Anxiety and Depression Scale; MCS, mental component score; NPI-D, Neuropsychiatric Inventory Distress Scale; PAC, Positive Aspects of Caring; PANAS, Positive and Negative Affect Schedule; PCS, Physical Component Score; QCPR, Quality of Caregiver–Patient Relationship; SF, short form.

**Table 4:** Outcomes for people with dementia after 12 months: means and differences adjusted for covariates and the other intervention

Measure	Data set	Missing (N=241)	CSP	No CSP	MD	95% CI of MD	Significance level	RYCT	No RYCT	MD	95% CI of MD	Significance level	Significance level of interaction
QoL-AD self-reported*	Original	101	37.85	37.93	−0.0820	(−1.70 to 1.56)	0.92	38.14	37.63	0.510	(−1.17 to 2.08)	0.58	0.51
	Imputed	0	36.43	36.59	−0.162	(−1.87 to 1.54)	0.85	36.86	36.16	0.702	(−1.05 to 2.45)	0.43	0.80
QoL-AD proxy reported*	Original	22	28.78	28.97	−0.200	(−1.44 to 1.05)	0.76	28.84	28.91	−0.0620	(−1.33 to 1.20)	0.92	0.02
	Imputed	0	28.50	28.49	0.050	(−1.21 to 1.31)	0.94	28.52	28.47	0.0660	(−1.17 to 1.30)	0.92	0.04
DEMQOL self-reported*	Original	103	96.65	94.11	2.54	(−0.67 to 5.74)	0.12	95.29	95.47	−0.173	(−3.35 to 3.00)	0.92	0.14
	Imputed	0	93.99	90.87	3.12	(−0.27 to 6.51)	0.07	92.19	92.67	−0.488	(−4.17 to 3.20)	0.80	0.20
DEMQOL proxy reported*	Original	24	92.41	95.59	−3.18	(−6.15 to −0.22)	0.04	93.99	94.00	0.00600	(−2.97 to 2.98)	0.99	0.08
	Imputed	0	92.22	95.11	−2.90	(−5.88 to 0.08)	0.06	93.49	93.84	−0.348	(−3.46 to 2.76)	0.83	0.20
EQ-5D self-reported utility*	Original	121	0.76	0.82	−0.07	(−0.17 to 0)	0.31	0.82	0.77	0.05	(−0.05 to 0.15)	0.24	0.22
	Imputed	0	0.75	0.84	−0.09	(−0.17 to −0.01)	0.11	0.80	0.79	0.03	(−0.07 to 0.13)	0.54	0.11
ED-5D self-reported general health*	Original	121	74.80	72.85	1.95	(−6.75 to 2.81)	0.51	72.95	74.70	1.74	(−6.91 to 2.52)	0.78	0.49
	Imputed	0	74.38	72.82	1.57	(−6.98 to 2.27)	0.72	72.48	74.72	2.24	(−6.24 to 3.25)	0.61	0.32
HADS total	Original	107	7.16	7.29	−0.132	(−1.87 to 1.60)	0.88	7.40	7.06	0.338	(−1.37 to 2.05)	0.70	0.21
	Imputed	0	9.27	9.42	−0.144	(−1.77 to 1.49)	0.86	9.49	9.21	0.280	(−1.18 to 1.74)	0.71	0.68
ADCS-ADL*	Original	68	40.70	42.79	−2.08	(−5.52 to 1.36)	0.24	40.18	43.31	−3.14	(−6.56 to 0.28)	0.07	0.02
	Imputed	0	35.30	37.47	−2.18	(−6.07 to 1.71)	0.27	35.16	37.61	−2.45	(−5.95 to 1.06)	0.17	0.07
NPI	Original	25	24.72	24.09	0.630	(−4.36 to 5.62)	0.81	25.43	23.38	2.05	(−2.98 to 7.07)	0.43	0.16
	Imputed	0	27.16	28.37	−1.20	(−6.64 to 4.23)	0.66	27.88	27.65	0.236	(−4.83 to 5.30)	0.93	0.40
QCPR total*	Original	104	60.50	59.66	0.841	(−1.22 to 2.90)	0.43	59.98	60.17	−0.196	(−2.22 to 1.83)	0.85	0.94
	Imputed	0	57.22	57.13	0.0880	(−2.71 to 2.88)	0.95	56.88	57.47	−0.598	(−2.61 to 1.41)	0.56	0.86

Covariates: baseline and MMSE scores, participant gender, kinship and centre (random effect).

* Higher scores are better.

ADCS-ADL, Alzheimer's Disease Cooperative Study—Activities of Daily Living; DEMQOL, quality of life for people with dementia; EQ-5D, EuroQol 5 dimensions; HADS, Hospital Anxiety and Depression Scale; MMSE, mini-mental state examination; NPI, neuropsychiatric inventory; QCPR, Quality of Caregiver–Patient Relationship; QoL-AD, Quality of Life in Alzheimer's Disease Scale.

For family carers, there was no significant main effect or interaction for the main outcome (health-related quality of life, SF12). For CSP versus ‘no CSP’, the difference in means was 0.52 points on the SF-12 (95% CI −1.28 to 2.32), and for RYCT versus ‘no RYCT’, the difference in means was 0.10 points on the SF-12 (95% CI −1.72 to 1.93). Neither was there any main effect or interaction for any secondary outcome, except for the quality of relationship, where imputed data showed significant benefit for CSP over ‘no CSP’ in the ‘absence of criticism and conflict’. The adjusted difference in means was 1.11 (95% CI 0.00 to 2.21, p=0.05). Comparison of the combined intervention with TAU (table 5) showed that by 12 months, CSP–RYCT had also improved carers' perceived quality of the caring relationship. The difference in means was 3.13 (95% CI 0.42 to 5.83, p=0.03).

**Table 5:** Combined intervention (CSP–RYCT) versus TAU: adjusted means and differences when main effect or interaction was significant at 12 months

		First follow-up						Second follow-up					
Measure	Data set	Missing	Combined	TAU	MD	95% CI of MD	Significance level	Missing	Combined	TAU	MD	95% CI of MD	Significance level
Carer QCPR absence of criticism and conflict*	Original	8	22.28	21.59	0.69	(−0.72 to 2.10)	0.34	14	22.39	20.76	1.63	(0.10 to 3.15)	0.04
	Imputed	0	21.97	21.09	0.88	(−0.85 to 2.34)	0.24	0	22.32	20.21	2.11	(0.54 to 3.69)	0.01
Carer QCPR total*	Original	8	55.57	53.43	2.14	(−0.22 to 4.50)	0.08	14	54.35	51.77	2.59	(−0.27 to 5.44)	0.08
	Imputed	0	54.98	52.51	2.47	(−0.02 to 4.97)	0.05	0	54.20	51.08	3.13	(0.42 to 5.83)	0.03
QoL-AD proxy*†	Original	7	30.73	31.42	−0.69	(−2.34 to 0.96)	0.41	12	29.68	29.88	−0.20	(−2.15 to 1.75)	0.84
	Imputed	0	30.49	31.22	−0.73	(−2.40 to 0.94)	0.39	0	29.68	29.88	−0.20	(−2.15 to 1.75)	0.84
DEMQOL proxy*†	Original	7	96.37	100.70	−4.31	(−8.19 to −0.44)	***0.03***	12	95.09	97.40	−2.31	(−6.57 to 1.94)	0.28
	Imputed	0	95.67	99.90	−4.23	(−9.08 to −0.38)	***0.03***	0	94.20	96.67	−2.48	(−6.98 to 2.02)	0.28
ADCS-ADL*†	Original	23	42.53	43.18	−0.65	(−4.70 to 3.39)	0.75	35	39.49	44.07	−4.58	(−9.29 to 0.13)	0.06
	Imputed	0	39.16	40.62	−1.46	(−5.47 to 2.54)	0.48	0	34.02	38.40	−4.39	(−10.20 to 1.43)	0.14

Covariates: baseline score, participant gender, kinship and centre (random effect).

*Higher scores are better.

†MMSE was also covariate in models for people with dementia.

ADCS-ADL, Alzheimer's Disease Cooperative Study—Activities of Daily Living; DEMQOL, quality of life for people with dementia; MMSE, mini-mental state examination; QCPR, Quality of Caregiver–Patient Relationship; QoL-AD, Quality of Life in Alzheimer's Disease Scale.

The results for people with dementia are more complex. There was no significant main effect for the main outcome (QoL-AD) at 12 months (CSP vs ‘no CSP’ difference in means −0.08 points, 95% CI −1.70 to 1.56; RYCT vs ‘no RYCT’ difference in means 0.51 points, 95% CI −1.17 to 2.08). However, the interaction between CSP and RYCT was significant (p=0.02), suggesting that the effectiveness of the combination might be different. The comparison of the combined intervention with TAU showed no significant effect on QoL-AD. Instead, CSP–RYCT adversely affected proxy-reported DEMQOL relative to TAU (difference in means −4.31, 95% CI −8.19 to −0.44, p=0.03 at the first follow-up). Similarly, CSP adversely affected proxy-reported DEMQOL (difference in means −3.18, 95% CI −6.15 to −0.22, p=0.04).

### Completers versus non-completers

We used Fisher's exact tests and Mann-Whitney U tests to compare baseline characteristics of those 241 pairs who completed the final follow-up with those 50 who withdrew before then. However, we found no significant difference in any demographic characteristics [carers' gender (p=0.74), age (p=0.12), ethnicity (p=0.67), marital status (p=0.62), level of education (p=0.76)]; dementia characteristics [type of dementia (p=0.53), neuropsychiatric symptoms (NPI, p=0.24), time since diagnosis (p=0.56)]; kin relations (spouses/partners vs non-spouses p=0.37); cohabitation (p=0.12); or psychological variables [health-related quality of life (SF12 MCS, p=0.67; SF12 PCS, p=0.82; EQ-5D utility, p=0.67); distress (NPI distress, p=0.24) or depression (HADS depression, p=0.65)].

### Intervention uptake and receipt

We offered 145 carers access to CSP—48 in the CSP only arm and 97 in the combined arm. Ninety (62%) took up the offer by meeting a CS at least once. Uptake was higher for CSP only (71%) than for the combined intervention (58%). On average carers who accepted CSP spent 17.8 hours with their CS over 13.1 sessions (median 12.5, range 1–40). This was higher in the CSP only arm (19.6 hours over 14.3 sessions) than in the combined arm (16.7 hours over 12.4 sessions).

We offered 194 carers access to an RYCT programme—97 in the RYCT only arm and 97 in the combined arm. In total, 112 (57%) attended at least 1 RYCT session, with little variation between the RYCT only (59%) and the RYCT component of the combined intervention (57%). Carers who attended at least 1 session attended a mean of 13.5 sessions of the possible maximum of 19, again with little variation between the RYCT only arm (13.1 sessions) and the combined arm (13.6 sessions).

Within the combined arm, 52 carers (54%) took up the Carer Support and reminiscence components with 82% taking up at least one of the interventions. More people took up CSP without RYCT (n=21) than RYCT without CSP (n=7).

Reasons for declining the RYCT intervention included: existing commitments (eg, carer's work and luncheon clubs for the person with dementia) especially if there was a perceived risk of jeopardising them; high level of impairment, cognitive or physical, in the person with dementia; and carer's dislike of group settings.

Despite these variations in the ‘doses’ of CSP and RYCT received, table 6 shows no association between outcomes and numbers of intervention sessions received. The coefficients are small and make little contribution to the model.

**Table 6:** Influence of treatment received on outcomes for participants with complete data at 12 months

	Number of RYCT sessions		Number of RYCT sessions attended by CS*		Number of CSP sessions	
	Coefficient†	95% CI	Coefficient†	95% CI	Coefficient†	95% CI
*Carer outcomes*						
SF12 MCS	−0.0365	(−0.180 to 0.107)	0.152	(−0.163 to 0.466)	0.0823	(−0.0365 to 0.201)
SF12 PCS	−0.115	(−0.262 to 0.0315)	0.220	(−0.0992 to 0.540)	0.0900	(−0.0311 to 0.211)
EQ-5D self-reported utility	−0.00376	(−0.00838 to 0.000850)	0.00660	(−0.00325 to 0.0165)	0.000135	(−0.00363 to 0.00390)
EQ-5D self-rated general health	−0.0606	(−0.418 to 0.296)	0.453	(−0.307 to 1.214)	0.0961	(−0.193 to 0.385)
HADS anxiety	−0.00616	(−0.0821 to 0.0698)	−0.107	(−0.270 to 0.0553)	0.00404	(−0.0581 to 0.0661)
HADS depression	0.00937	(−0.0602 to 0.0789)	−0.145	(−0.294 to 0.00518)	−0.0253	(−0.0827 to 0.0321)
HADS total	0.00790	(−0.125 to 0.141)	−0.252	(−0.538 to 0.0341)	−0.0256	(−0.135 to 0.0839)
PANAS positive affectivity	0.0200	(−0.0957 to 0.136)	0.157	(−0.0915 to 0.405)	−0.150	(−0.110 to −0.0795)
COPE PAC	0.0103	(−0.0308 to 0.0514)	0.0407	(−0.0470 to 0.128)	0.0398	(0.00580 to 0.0737)
Carer distress NPI-D	0.0351	(−0.142 to 0.212)	−0.0494	(−0.422 to 0.324)	0.0732	(−0.0755 to 0.222)
Personal growth	−0.000814	(−0.0476 to 0.0460)	−0.00282	(−0.105 to 0.100)	0.00681	(−0.0316 to 0.0452)
QCPR warmth	−0.0222	(−0.116 to 0.0719)	0.0223	(−0.179 to 0.223)	0.0522	(−0.0260 to 0.131)
QCPR absences of criticism and conflict	−0.0116	(−0.0946 to 0.0715)	−0.0229	(−0.202 to 0.156)	−0.0368	(−0.106 to 0.0324)
QCPR Total	−0.0329	(−0.184 to 0.118)	−0.0142	(−0.338 to 0.309)	0.0183	(−0.108 to 0.144)
Loneliness	−0.00569	(−0.0450 to 0.0336)	0.0148	(−0.0692 to 0.0989)	0.00345	(−0.0286 to 0.0356)
*Person with dementia outcomes*						
QoL-AD self-reported	0.0730	(−0.0632 to 0.209)	−0.0483	(−0.381 to 0.284)	−0.0295	(−0.135 to 0.0755)
QoL-AD proxy reported	0.0351	(−0.0650 to 0.135)	−0.0531	(−0.273 to 0.167)	−0.0933	(−0.175 to −0.0115)
DEMQOL self-reported	0.0891	(−0.186 to 0.364)	0.232	(−0.523 to 0.986)	−0.0891	(−0.299 to 0.121)
DEMQOL proxy reported	−0.0879	(−0.322 to 0.146)	−0.00380	(−0.497 to 0.489)	−0.0272	(−0.220 to 0.166)
HADS anxiety	0.0218	(−0.0649 to 0.108)	−0.0528	(−0.289 to 0.184)	0.0126	(−0.0537 to 0.0790)
HADS depression	−0.0331	(−0.121 to 0.0552)	−0.0101	(−0.253 to 0.233)	0.0103	(−0.0576 to 0.0783)
HADS total	−0.0182	(−0.166 to 0.129)	−0.0591	(−0.461 to 0.343)	0.0175	(−0.0959 to 0.131)
ADCS-ADL	0.185	(−0.103 to 0.473)	−0.261	(−0.894 to 0.373)	−0.213	(−0.442 to 0.0162)
QCPR warmth	0.0643	(−0.0366 to 0.165)	−0.118	(−0.400 to 0.163)	0.0163	(−0.0618 to 0.0944)
QCPR absence of criticism and conflict	0.158	(0.0514 to 0.265)	−0.204	(−0.495 to 0.0833)	0.0147	(−0.0690 to 0.0985)
QCPR total	−0.0166	(−0.154 to 0.121)	0.161	(−0.126 to 0.447)	−0.101	(−0.210 to 0.00847)

*Combined intervention.

†Coefficients represent the mean change in outcome with change in number of sessions attended when all other variables in the model are constant; negative coefficients show that outcomes deteriorate as attendance increases.

ADCS-ADL, Alzheimer's Disease Cooperative Study—Activities of Daily Living; COPE, Carers of Older People in Europe; NPI-D, Neuropsychiatric Inventory Carer Distress Scale; CS, carer supporter; DEMQOL, quality of life for people with dementia; EQ-5D, EuroQol 5 dimensions; HADS, Hospital Anxiety and Depression Scale; MCS, mental component score; PAC, Positive Aspects of Caring; PANAS, Positive and Negative Affect Schedule; PCS, physical component score; QCPR, Quality of Caregiver–Patient Relationship; QoL-AD, Quality of Life in Alzheimer's Disease Scale; SF, short form.

## Discussion

Through this trial, we sought to answer the question ‘do peer support or reminiscence, together or separately, enhance quality of life for family carers and people with dementia?’ There was no indication from the trial to suggest that either peer support or reminiscence resulted in any measurable benefit.

Primary analysis 12 months after randomisation showed no benefit to family carers of peer support or reminiscence therapy on their many outcome measures; the only exception is that peer support, separately and combined with reminiscence therapy, improved carers' perceived relationship with the person with dementia. Similarly, there was very little benefit to people with dementia on their many outcome measures. The only exception is that carers allocated to peer support rated quality of life significantly lower for the people with dementia. The corresponding people with dementia reported higher, but not significantly higher, quality of life. This may reflect the known tendency of people with dementia to rate their quality of life more highly than do their relatives;^27^ however, the carer versus person with dementia rating discrepancy was not evident in any of the other comparisons.

Our lack of findings are in keeping with many other randomised controlled trials of psychological interventions for family carers of people with dementia where no measurable effect has been found for popular interventions.

### Strengths and limitations

We drew participants from a wide range of community settings and included those already embedded in services and new users of those services. We adopted broad inclusion criteria reflecting the wide range of UK settings. Both interventions benefitted from well-developed manuals and training for all providers. The follow-up interviewers were blind to participants' allocations, although participants inevitably knew which interventions they had received. We met our recruitment and retention targets, though the intervention groups retained slightly more (83%) than the control group (77%). There were no harms associated with the interventions. Of the 159 serious adverse events recorded during the trial, 3 were attributable to RYCT; however, none led to withdrawal.

Factorial trial designs are attractive in yielding ‘two trials for the price of one’. We overcame the challenge of running overlapping interventions by sequential randomisation procedures. However, we adapted the original trial protocol for each intervention to create a protocol that could deliver both. For example, the eligibility criteria for reminiscence therapy usually exclude people with dementia with agitation or severe cognitive or physical impairment. Nevertheless, we enrolled them as eligible for home-based peer support. Where possible, we adapted interventions accordingly, for example by hiring specialist transport or allowing carers to attend reminiscence sessions without their relatives. As we could not accommodate all such needs within the interventions, however, recruitment fell, but not below our targets.

Factorial designs are less easy to interpret. They generate four groups, of which three receive interventions, but only one gets TAU. However, the main analyses reduce these four groups to two: to evaluate peer support, analysis compares those allocated to support with or without reminiscence therapy with those not so allocated, and to evaluate reminiscence therapy, analysis compares those allocated to reminiscence with or without peer support with those not so allocated. These analyses assume that peer support and reminiscence do not interact in the sense that one potentiates or weakens the other. However, three of many interactions we tested were significant. Hence, we interpret the corresponding non-significant main effects with caution. Though we addressed this issue by comparing the combined intervention with TAU, this comparison does not have as much power.

### Differences between this and other studies

*Intervention:* In this trial, we refined both interventions better to meet the needs of family carers, using past carers of people with dementia rather than lay befrienders increased rapport between participating carers and their supporters and reminiscence therapy put more emphasis on carer support and education than in the REMCARE trial.

*Design:* We adopted a factorial design capable of evaluating both interventions simultaneously, and testing whether they potentiate each other.

*Population:* We recruited more non-white carers and people with dementia than previous dementia care trials had performed in the UK. There were significant differences between carers of different ethnic backgrounds, with South Asians reporting most distress and African Caribbean carers least.

*Uptake:* This varied between groups. Uptake of carer interventions in trials is often low, which threatens trials analysed by treatment allocated. In contrast, the success of the START trial was due at least in part to very good uptake by carers.^1^

*Findings:* The paucity of benefit to carers is consistent with previous trials of befriending^2^ and reminiscence.^4^ However, we did not see the REMCARE finding of *increased* anxiety in carers receiving reminiscence therapy.^4^ Instead, our qualitative evidence^28^ ^29^ aligns with other evidence that peer support and reminiscence therapy are valued and enjoyable activities.^30^ Previous carer intervention research has received criticism for not studying impact on people with dementia.

### Unanswered questions and future research

Peer support and reminiscence therapy are attractive in principle, but neither has yet generated evidence of effectiveness. By adopting a factorial design, we sought to test whether they potentiate each other; however, this was also ineffective. Interventions targeting people with dementia and their carers have gained in popularity. The findings of this research raise questions about how best to balance the needs of the family carer and his or her relative with dementia. It also reinforces the need to study the impact of interventions on both parties.

## Conclusions

Despite enhancing the carer support component of peer support and reminiscence, this trial confirms previous findings that neither intervention is effective. The quantitative results are at odds with findings from qualitative studies of the same interventions. Research in this field should now seek to identify which carers and people with dementia can benefit from which psychosocial interventions.
